# Reduced starch granule number per chloroplast in the *dpe2/phs1* mutant is dependent on initiation of starch degradation

**DOI:** 10.1371/journal.pone.0187985

**Published:** 2017-11-20

**Authors:** Irina Malinova, Joerg Fettke

**Affiliations:** Biopolymer analytics, University of Potsdam, Potsdam-Golm, Germany; Universidade Federal de Vicosa, BRAZIL

## Abstract

An Arabidopsis double knock-out mutant lacking cytosolic disproportionating enzyme 2 (DPE2) and the plastidial phosphorylase (PHS1) revealed a dwarf-growth phenotype, reduced starch content, an uneven distribution of starch within the plant rosette, and a reduced number of starch granules per chloroplast under standard growth conditions. In contrast, the wild type contained 5–7 starch granules per chloroplast. Mature and old leaves of the double mutant were essentially starch free and showed plastidial disintegration. Several analyses revealed that the number of starch granules per chloroplast was affected by the dark phase. So far, it was unclear if it was the dark phase *per se* or starch degradation in the dark that was connected to the observed decrease in the number of starch granules per chloroplast. Therefore, in the background of the double mutant *dpe2/phs1*, a triple mutant was generated lacking the initial starch degrading enzyme glucan, water dikinase (GWD). The triple mutant showed improved plant growth, a starch-excess phenotype, and a homogeneous starch distribution. Furthermore, the number of starch granules per chloroplast was increased and was similar to wild type. However, starch granule morphology was only slightly affected by the lack of GWD as in the triple mutant and, like in *dpe2/phs1*, more spherical starch granules were observed. The characterized triple mutant was discussed in the context of the generation of starch granules and the formation of starch granule morphology.

## Introduction

Transitory starch metabolism is a central process in the plant life cycle. However, many aspects of the synthesis and degradation of starch granules remain obscure. Thus, neither the physicochemical mechanism nor the proteins involved in the initiation and formation of the starch granules have been identified. Arabidopsis wild-type chloroplasts contain a strictly regulated number of starch granules, with 5–7 granules per chloroplast [1]. However, a significant alteration of the number of starch granules per chloroplast was observed in plants lacking soluble starch synthase 4 (SS4; [2]). In this mutant, only one starch granule per chloroplast was detected. Surprisingly, analysis of double mutant *dpe2-1/phs1a* lacking DPE2 (disproportionating enzyme 2), a cytosolic enzyme involved in starch related maltose metabolism during starch degradation [3], [4], [5] and PHS1 (plastidial phosphorylase; [6]) also revealed one starch granule per chloroplast, at least in young leaves [7]. Furthermore, a triple mutant lacking DPE2, PHS1, and SS4 also revealed one starch granule per chloroplast. Thus, it was concluded that the synthesis of this single starch granule was independent of the pathways absent in the *ss4* and *dpe2-1/phs1a* mutants [8]. However, the number of starch granules per chloroplast in *dpe2-1/phs1a* was dependent on the length of the light phase. Thus, under short-day conditions, the number of starch granules per chloroplast was mainly one, whereas under long-day conditions, the starch granule number increased to up to four granules per chloroplast [8]. However, when *dpe2-1/phs1a* was grown under continuous light, the number of starch granules per chloroplast was 5–7, which is the same as in the wild type [7]. Interestingly, in this regard, *dpe2-1/phs1a* and *dpe2-1/phs1a/ss4* were different. Unlike *dpe2-1/phs1a*, in the triple mutant, the starch granule number remained reduced (zero to four granules per chloroplast) even under continuous light [8]. Furthermore, starch granule morphology was also affected. *Dpe2-1/phs1a* contained larger and more spherical starch granules compared to the wild type and the corresponding single parental mutant lines. Moreover, *dpe2-1/phs1a/ss4* revealed large and nearly perfectly spherical starch granules [8].

A biochemical explanation for the observed reduction in the number of starch granules per chloroplast in the mutants remains obscure. No referable metabolic alteration was observed [7], [8]. However, it was concluded that the regulation of the number of starch granules per chloroplast is associated with the dark phase, particularly with starch degradation [8].

During starch depletion the main degradation product maltose is exported from the chloroplast by MEX1 (**M**altose **ex**porter 1; [9]) into the cytosol. Since the triple mutant *dpe2-5/phs1b/mex1* revealed a reduced number of starch granules per chloroplast (zero to two), it was concluded that the maltose catabolism in the cytosol does not have an impact on the number of starch granules per chloroplast [8]. Furthermore, *mex1/phs1b* accumulated maltose but showed no differences in the number of starch granules per chloroplast compared to wild type [7]. Thus, a simple connection between maltose metabolism and the observed reduction in the number starch granules per chloroplast in *dpe2-1/phs1a* can be excluded.

To verify the assumption that starch degradation influences the number starch granules per chloroplast in the *dpe2-1/phs1a* mutant, we generated a mutant additionally lacking the initial enzyme in starch degradation, namely glucan, water dikinase (GWD; *sex1-8* [10], [11], [12]). In the current model of starch degradation, GWD is involved in the starch phosphorylation/ de-phosphorylation cycle at the starch granule surface [13], [14], and the lack of GWD results in a massive starch-excess phenotype and a nearly-total inhibition of starch breakdown [15]. The generated triple mutant *dpe2-1/phs1a/sex1-8* was analysed with a focus on the number of starch granules per chloroplast.

## Materials and methods

### Plant materials and growth conditions

Knockout lines *dpe2-5*, *phs1b*, *sex1-8*, and *dpe2-5/phs1b* in the Col-0 background have been described previously [3], [7], [8], [12]. *Phs1b/sex1-8* and *dpe2-5/sex1-8* were generated by crossing the respective homozygous single mutants and self-pollination of the F1 generation. The *dpe2-5/phs1b/sex1-8* mutant was generated by crossing *dpe2-5/phs1b* and *sex1-8* and self-pollination of the F1 generation.

To screen the F2 generation, DPE2 and phosphorylases activities were detected by native gels [16] and zymograms [7]. Analysis of the GWD protein was performed by SDS-PAGE, western blotting, and immunodetection as described by Mahlow *et al*. [12]. Plants were grown in a light-dark regime (12 h light, 20°C, 110 μmol m^-2^s^-1^; 12 h dark, 16°C; relative humidity throughout the light-dark cycle was kept at 60%) unless otherwise stated.

### Scanning electron microscopy (SEM) and transmission electron microscopy (TEM)

SEM analysis was performed as described by Mahlow *et al*. [12]. Starch granule diameters were estimated using SmartTiff Software (Zeiss). TEM samples were analysed as described by Malinova *et al*. [7].

### Starch analyses

Starch was quantified according to Malinova *et al*. [7]. Native starch granules were isolated as described by Malinova *et al*. [8].

### Isoamylase treatment of native starch granules

Isoamylase digestion of heat solubilized starch granules was performed as described by Mahlow *et al*. [7]. The chain length distribution was analysed by capillary electrophoresis equipped with laser-induced fluorescence detection (CE-LIF) as described by Malinova *et al*. [7].

## Results

### Additional elimination of GWD in the *dpe2-5/phs1b* background caused recovery of the starch-excess phenotype and improved growth

**Fig 1:**
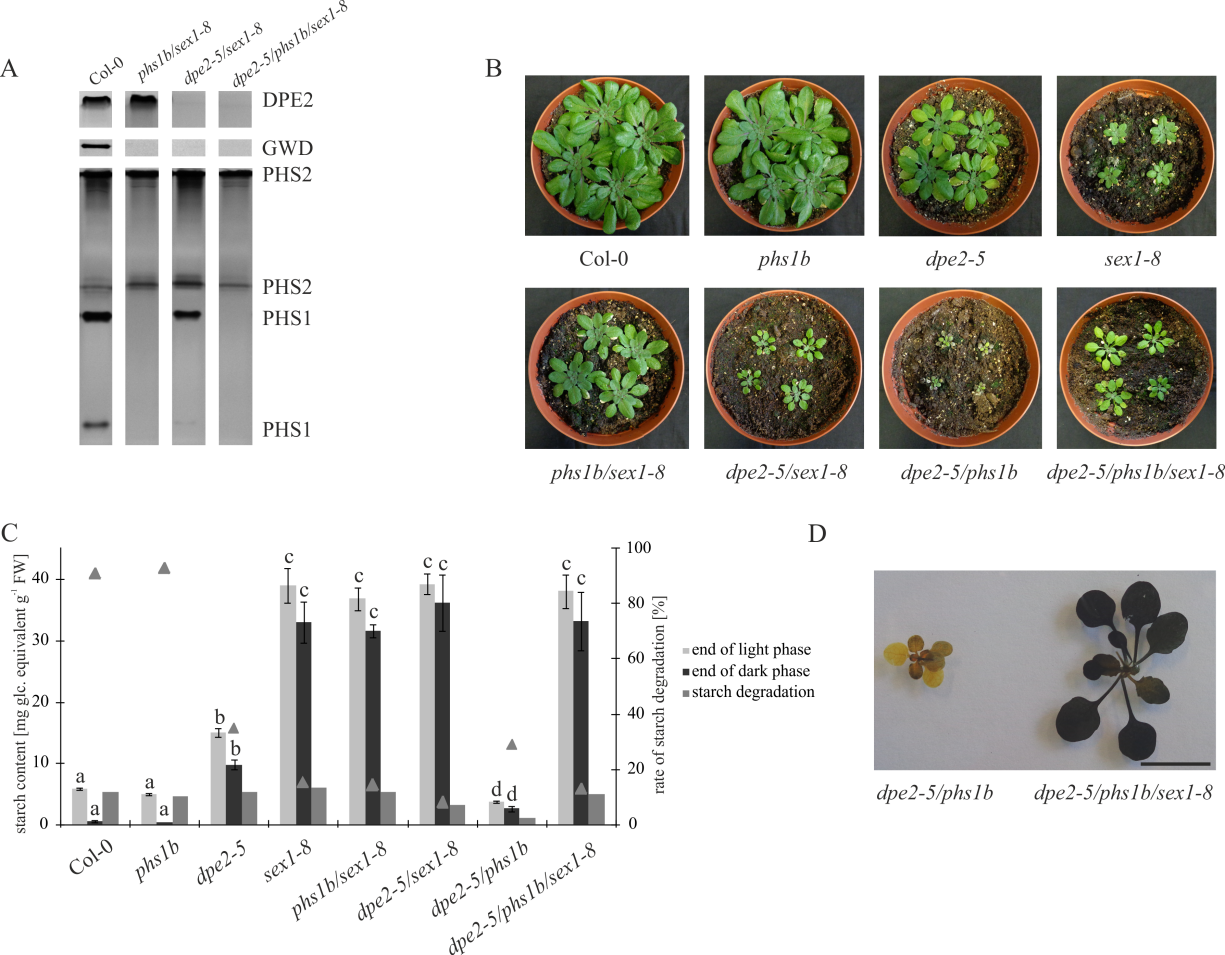
Growth phenotype and carbohydrate analyses of mutants and parental lines. (A) Screening of generated double and triple mutants. One leaf (comparable sizes) from each plant was harvested and proteins were isolated in 120 μl of extraction buffer. A total of 25 μl of each was loaded per lane. DPE2 and PHS proteins were separated by discontinuous native PAGE, and their activities were detection. For GWD detection, crude extracts were separated by SDS-PAGE followed by western blot and immunodetection against GWD. (B) Growth phenotype of the generated mutants and wild type. Plants were six -weeks-old. (C) Starch content. All values are mean ± SD (n = 3–4 replicates from a mixture 4–10 plants each). Letters indicate samples that were not significantly different (P<0.05) according to one-way ANOVA with Holm-Sidak post hoc testing. The third bar indicates the level of starch degraded by the end of the night phase (starch content at the end of the day minus starch content at the end of the night). Triangles represent the amount of starch degraded at the end of the night [%]. (D) Semi quantitative starch determination. Plants were harvested at the end of the light phase (11 h in the light), decolorized in hot 80% (v/v) ethanol, and stained with iodine solution.

Triple knock-out mutants lacking DPE2, PHS1, and GWD were generated by crossing *dpe2-5/phs1b* [8] with *sex1-8* [12]. The absence of DPE2 and PHS1 was confirmed by native PAGE and subsequent activity staining. The lack of GWD was confirmed by immunoblotting using an antibody against GWD (Fig 1A).

Plants were cultivated under a 12 h light/12 h dark regime. *Dpe2-5* and *sex1-8* showed a smaller shoot size compared to the wild type, as described (Fig 1B; [3], [12]). No difference in growth was observed for *phs1b* compared to the wild type. However, *phs1b/sex1-8* showed an intermediate growth phenotype in comparison to corresponding parental lines, whereas *dpe2-5/sex1-8* revealed a smaller plant size compared with *dpe2-5* and *sex1-8*. *Dpe2-5/phs1b* showed the strongest dwarf phenotype as reported previously [8]. Interestingly, *dpe2-5/phs1b/sex1-8* exhibited a larger shoot size compared to *dpe2-5/phs1b*, as well as to *sex1-8* and *dpe2-5/sex1-8* (Fig 1B).

With regard to starch content, *dpe2-5* and *sex1-8* revealed a starch-excess phenotype, although the starch content in *sex1-8* was much higher (Fig 1C). This is in agreement with the current starch degradation model, in which GWD is a key enzyme for the initiation of starch degradation, whereas DPE2 acts downstream and is involved in metabolism of starch-related maltose in the cytosol.

All mutants lacking GWD exhibited a starch-excess phenotype. Thus, the triple mutant *dpe2-5/phs1b/sex1-8* also showed a similar starch-excess phenotype as *sex1-8*, *phs1b/sex1-8* and *dpe2-5/sex1-8*, whereas *dpe2-5/phs1b* showed a decrease in starch content compared to the parental *dpe2-5* line (Fig 1C). All mutants lacking GWD activity revealed a very low starch turn over as the amount of starch degraded by the end of the night was approximately 7–15% (starch content at the end of the light = 100%). *Dpe2-5* and *dpe2-5/phs1b* also showed a decrease in starch degradation; however in this case, approximately 30% of the starch was degraded. Furthermore, for double mutants lacking DPE2 and PHS1, an unequal starch distribution was described; thus, starch was only detected in young leaves, while mature leaves were essentially starch free [7], [8]. In contrast, the additional lack of GWD resulted in massive starch accumulation in all leaves (Fig 1D).

### In *dpe2-5/phs1b/sex1-8* the starch granule formation was recovered, as the detected number of starch granules per chloroplast was similar to wild type

**Fig 2:**
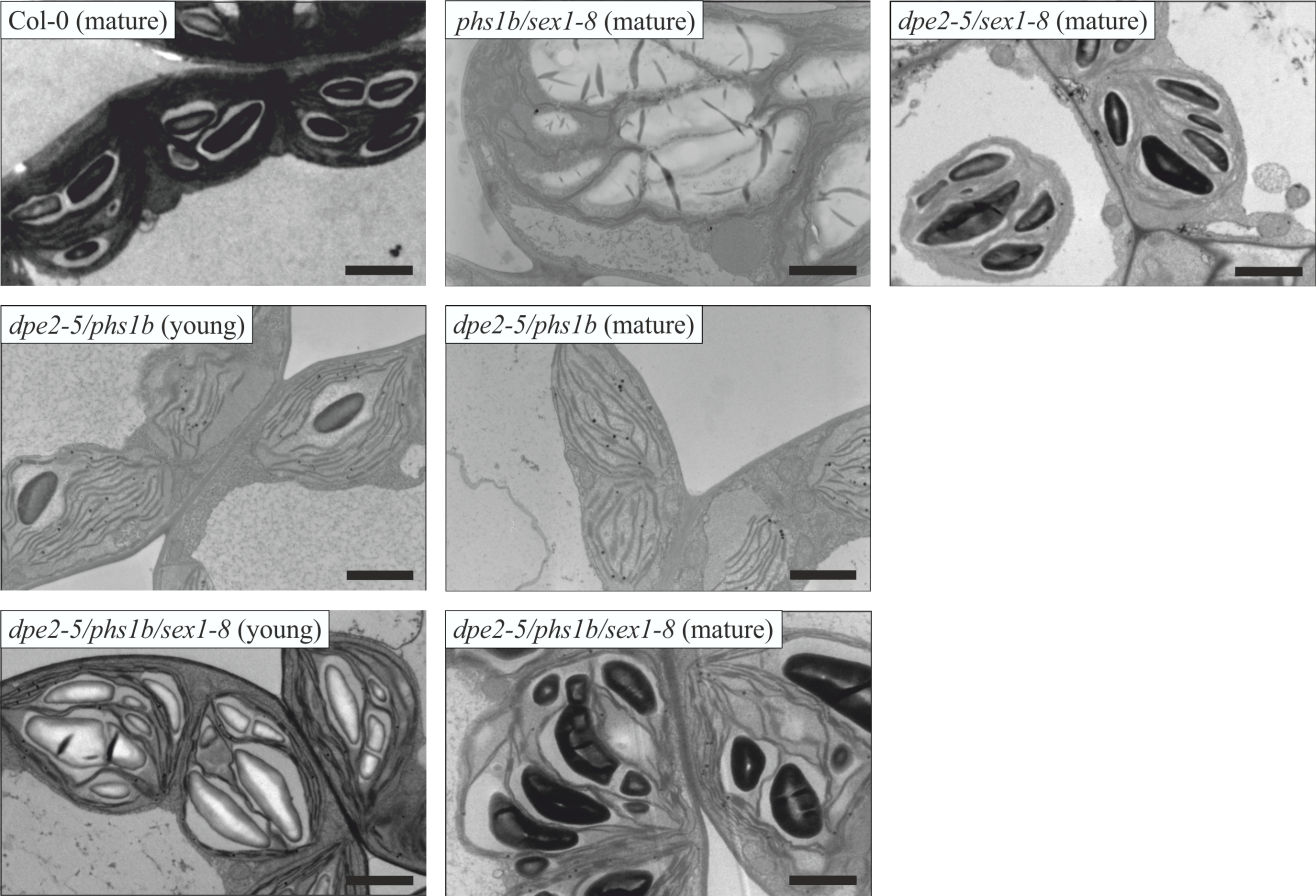
Transmission electron microscopy analysis of generated lines. Six-week-old plants were harvested in the middle of the light phase. Bars = 2 μm.

*Dpe2-5/phs1b* (Col-0 background) as well as *dpe2-1/phs1a* (Ws-0 background) showed a decrease in the number of starch granules per chloroplast. Generally, mainly one starch granule was detected per chloroplast in young leaves, whereas mature leaves revealed no starch and disintegration of the chloroplasts [7], [8]. As we assumed that the regulation of the number of starch granules per chloroplast in these mutants is connected to ongoing starch degradation, we subjected *dpe2-5/phs1b/sex1-8* to transmission electron microscopy (TEM). The chloroplasts of young leaves of *dpe2-5/phs1b* contained one starch granule per chloroplast, as previously described (Fig 2; [8]). However, in the triple mutant, the number of starch granules was massively increased to 5–7 starch granules per chloroplast, similar to wild type (Fig 2). The observed number of starch granules was independent of the age of the leaf, since 5–7 starch granules per chloroplasts were also detected in mature leaves (Fig 2). Furthermore, no disintegration of the chloroplasts was observed for the triple mutant in contrast to *dpe2-5/phs1b* (Fig 2).

In *dpe2-5/sex1-8* and *phs1b/sex1-8*, the number of starch granules per chloroplast, at 5–7, was similar to wild type and to the single parental lines (Fig 2, [7], [8]).

### The additional lack of GWD in the background of *dpe2-5/phs1b* has little impact on starch granule morphology

For mutants lacking DPE2 or SS4 rounder starch granules were observed compared to the typical flat, discoid, wild-type starch granules [7], [8], [17]. The starch granules of *dpe2-1/ss4* as well as those of *dpe2-1/phs1a/ss4* revealed a near-perfect spherical form [8]. All these mutants contained only one starch granule per chloroplast, and thus we concluded that a reduction in starch degradation, detected in all of these mutants, and the additional decrease in the starch granule number influences starch granule morphology in the formation of spherical granules. In contrast, *sex1-8* contained deformed, thin, and uneven granules [12].

**Fig 3:**
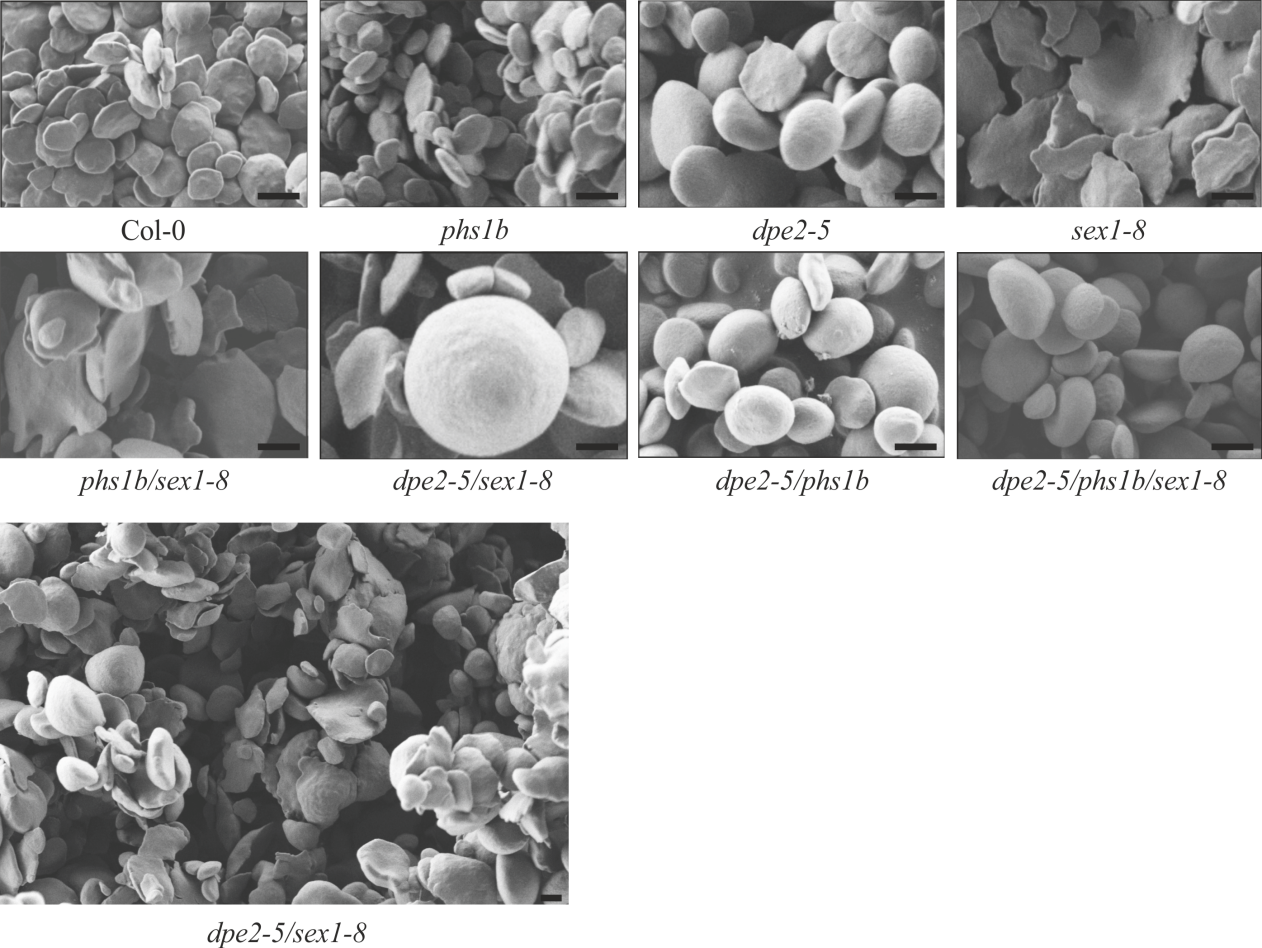
Scanning electron microscopy analysis of native starch granules isolated from leaves of the various mutants and wild type. Starch granules were isolated from the leaves of six-week-old plants harvested at the end of the light phase. Bars = 2 μm.

The newly generated mutants, *dpe2-5/sex1-8* and *phs1b/sex1-8*, revealed a starch granule morphology similar to *sex1-8* (Fig 3). However, starch granules isolated from *dpe2-5/sex1-8* were more heterogeneous; in addition to granules resembling the *sex1-8* morphology, a few spherical granules were also detected. In contrast, *dpe2-5/phs1b/sex1-8* revealed typical spherical starch granules, indistinguishable from *dpe2-5/phs1b* (Fig 3). Thus, the starch granule morphology in this mutant was not dominated or influenced by the lack of GWD, in contrast to the double mutants, *phs1b/sex1-8* and *dpe2-5/sex1-8*. However, the lack of GWD pre-dominates the observed starch granule particle size. Thus, all mutants lacking GWD exhibited bigger starch granules of approximately 5–6.5 μm, whereas wild type and *dpe2-5/phs1b* contained starch granules of 1.8 ± 0.3 μm and 3.5 ± 0.7 μm, respectively (Table 1).

When comparing the inner structures of the starch isolated from *dpe2-5/phs1b* and *dpe2-5/phs1b/sex1-8*, differences were observed. Shorter glucan chains with DP8-9 and 14–15 were increased in both mutants, while longer chains (DP26-35) were decreased compared to wild type.

**Fig 4:**
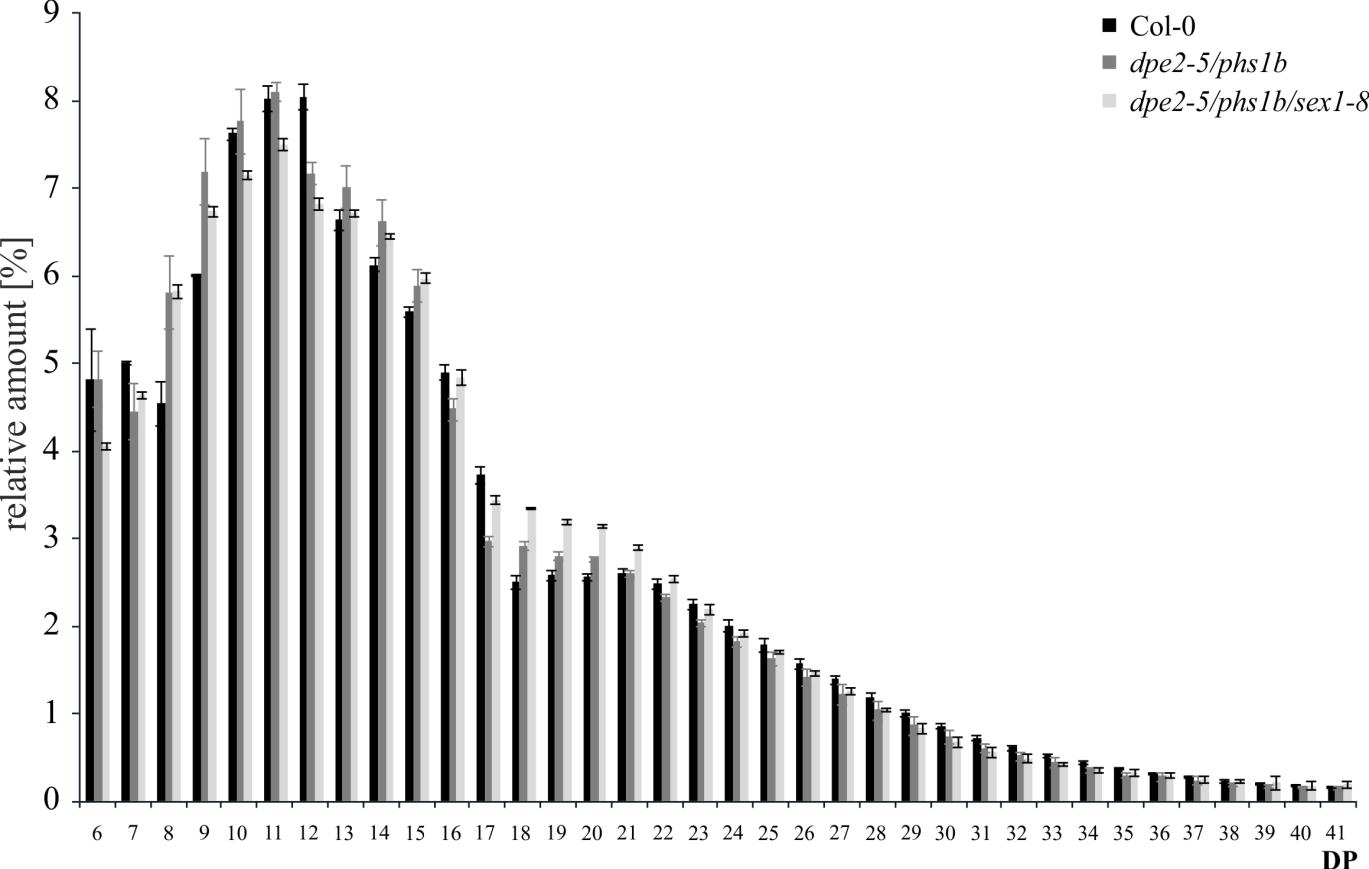
Chain length distribution (CLD) pattern of starch granules isolated from *dpe2-5/phs1b*, *dpe2-5/phs1b/sex1-8*, and Col-0. CLD profiles of isolated starch granules after heat solubilization and subsequent isoamylase treatment. All values are mean ± SD (n = 3). DP- degree of polymerization.

However, when comparing the mutants, the strongest alteration was observed for glucan chains with a degree of polymerization (DP) of 18–21. The number of glucan chains were further increased in the triple mutant (Fig 4).

## Discussion

This work described a newly generated triple mutant lacking DPE2, PHS1, and GWD. *Dpe2-5/phs1b/sex1-8* allowed us to prove that the dark phase *per se* or, as hypothesized, the process of starch degradation is responsible for the decrease in number of starch granules per chloroplast in *dpe2/phs1*. *Sex1-8* mutant lacking the activity of GWD demonstrated a very minor starch turn over rate through the light/dark cycle [12]. Furthermore, this mutant showed one of the most significant decreases in starch degradation described so far. Thus, even when *sex1-8* was transferred to an elongated dark phase, e.g., 24 h or 48 h of darkness, the starch content was high, and very little degradation of starch was detected [13]. In contrast, wild type Arabidopsis was essentially starch free after 12 h in the dark. Therefore, we chose this mutant for the generation of the triple mutant.

### The number of starch granules per chloroplast in *dpe2/phs1* is dependent on ongoing starch degradation

*Dpe2-5/phs1b/sex1-8* showed improved plant growth compared to *dpe2-5/phs1b* and a typical starch distribution, thus all leaves contained starch (Fig 1C and 1D). Furthermore, in contrast to *dpe2-5/phs1b*, the triple mutant showed a starch-excess phenotype (Fig 1C). For all mutants lacking GWD, a small amount of starch, approximately ten percent, was degraded during the dark phase (Fig 1C). Furthermore, with regard to the starch content at the end of both the light and dark phases, no significant alterations were observed for any mutants lacking GWD. Similarly, when examining the number of starch granules per chloroplast, the newly generated double-mutant controls *dpe2-5/sex1-8*, and *phs1b/sex1-8*, as well as the triple mutant *dpe2-5/phs1b/sex1-8*, all contained 5–7 starch granules per chloroplast, as described for the wild type (Fig 2). Thus, the lack of GWD in the background of *dpe2/phs1* resulted in an increased number of starch granules per chloroplast to a number indistinguishable from wild type.

**Fig 5:**
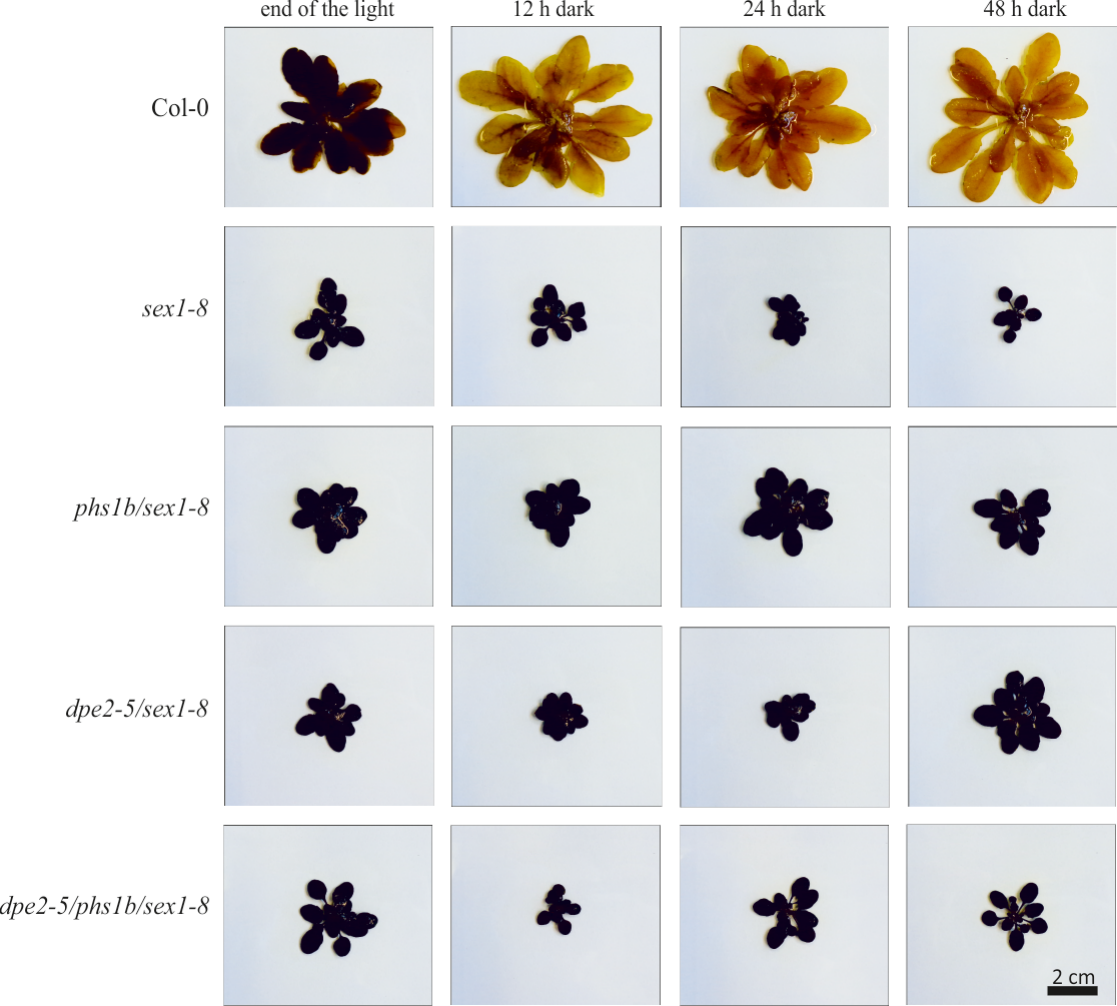
Semi-quantitative starch determination in plants under prolonged dark conditions. Plants were grown under 12 h light/12 h dark conditions for 5 weeks.

In principle, the lack of GWD has two effects on starch. First, no C6-phosphate esters were introduced. Second, not only does the lack of this phosphate ester have an impact on starch degradation, it also influences starch syntheses [12], [18][13]. It could be speculated that C6-phosphorylated glucans, generated during starch degradation at night, influence the formation of starch granules. Thus, for example, phosphorylated and non-phosphorylated glucans could have different effects on the formation/initiation of starch granules. Interestingly, in mutants lacking GWD, an increase in the number of starch granules per chloroplast was observed [12]. However, this mechanistic background is highly unlikely, as there was no detectable accumulation of phosphoglucans in *dpe2/phs1* mutants [8]. Furthermore, it is hard to assume that phosphoglucans play an important role in this process since, in principle, they are easily degraded to glucans by various dephosphorylating enzymes in the chloroplast stroma. Thus, the inhibition of starch degradation is more likely to be responsible for the observed increase in the starch granule number. Although for all mutants lacking GWD, a small but detectable amount of starch degradation was observed (Fig 1C), for *sex1-8*, a total degradation of starch was impossible even during an elongated dark phase (Fig 5). This was also tested in *dpe2-5/phs1b/sex1-8* and the corresponding double mutants (Fig 5). A further degradation of starch was not detected.

Thus, it is highly likely that ongoing starch degradation is necessary for the observed decrease in number of starch granules per chloroplast, although a direct link between them is still obscure. A step-wise analysis of several triple mutants in *dpe2/phs1* background, which lacks enzymes related to starch degradation downstream of GWD, should allow us to identify the link. However, several double mutants (*phs1/sex1-8*; *mex1/phs1*, *dpe2-5/sex1-8*, see also [7], [8]) point to a special combination of missing enzymes in *dpe2/phs1*. Thus, *dpe2/phs1* is unique in the reduction in the number of starch granules per chloroplast. Since DPE2 has repeatedly been shown to be a cytosolic enzyme [3]; [5]), a connection between plastidial starch degradation and cytosolic DPE2 is expected. Whether or not the catalytic activity of DPE2 is necessary or for example, the structural function of the DPE2 protein is involved is unclear. However, when considering the catalytic function of DPE2 in this context, the metabolism of cytosolic heteroglycans [5] as well as glucose phosphate pathways between the chloroplast and the cytosol [19] should be the focus of further analyses.

### The inner starch granule structure and the granule morphology in *dpe2/phs1* are only slightly affected by an additional loss of GWD

When analysing the starch granules by SEM, Col-0, and *phs1b* exhibited the typical flat discoid Arabidopsis starch granules. *Dpe2* and *dpe2-5/phs1b* showed rounder starch granules, as described [7], [8], whereas for *sex1-8*, the typical deformed, thin and uneven granules were detected [12]. Furthermore, starch granules isolated from *phs1b/sex1-8* and *dpe2-5/sex1-8* also showed more deformed, thin and uneven starch granules. Although for the latter, several roundish and relatively big starch granules were observed (Fig 3).

Thus, the starch morphology is massively affected by the lack of GWD in these two mutants. In contrast, the starch granules isolated from the triple mutant revealed no obvious differences in their morphologies compared to *dpe2-5/phs1b* (Fig 3). Similarly, the inner structure of the starch granules isolated from *dpe2-5/phs1b* and *dpe2-5/phs1b/sex1-8* revealed only small differences (Fig 4). Thus, GWD plays a minor role in the generation of the inner starch structure and morphology in *dpe2-5/phs1b*, whereas in the other mutants it has a dominant effect. However, the lack of GWD has an important effect on the starch granule size. Thus, for all mutants lacking GWD, the starch size is massively increased to 5–6.5 μm (Table 1). However, in this regard, the uneven and flat shape of the starch granules must be considered. Furthermore, for all mutants, the massive starch accumulation observed in the absence of GWD (Fig 1C) was accompanied by an increase in the starch granule size, whereas the starch morphology was specifically affected in the various mutants (Fig 3).

**Table 1:** Estimated sizes of native starch granules. Values represent average ± SD (n = 27–35). * Granules are deformed; the largest part of the granule was measured. Letters indicate samples that were not significantly different (p<0.05) according to one-way ANOVA with Holm-Sidak posthoc testing.

Genotype	Size (diameter) [μm]
Wt	1.8 ± 0.3^**a**^
sex1-8*	6.5 ± 2.1^**b**^
phs1b/sex1-8*	5.0 ± 1.4^**c**^
*dpe2-5/phs1b*	3.5 ± 0.7^**d**^
*dpe2-5/phs1b/sex1-8*	6.0 ± 1.0^**b**^
*dpe2-5/sex1-8*	6.1 ± 2.1^**b**^

In summary, by generating *dpe2-5/phs1b/sex1-8*, we were able to show that the reduced number of starch granules per chloroplast in *dpe2/phs1* was dependent on ongoing starch degradation in the dark, whereas the starch morphology, and thus the formation of more spherical starch granules, was only slightly influenced by early inhibition of starch degradation.
